# Graphene-Modified 3D Copper Foam Current Collector for Dendrite-Free Lithium Deposition

**DOI:** 10.3389/fchem.2019.00748

**Published:** 2019-11-27

**Authors:** Juan Yu, Yangyang Dang, Maohui Bai, Jiaxin Peng, Dongdong Zheng, Junkai Zhao, Linbo Li, Zhao Fang

**Affiliations:** ^1^School of Metallurgical Engineering, Xi'an University of Architecture and Technology, Xi'an, China; ^2^Shaanxi Province Metallurgical Engineering and Technology Research Centre, Xi'an, China; ^3^School of Metallurgy and Environment, Central South University, Changsha, China

**Keywords:** Li metal anode, rGO@Cu foam, Li dendrite, current collector, LiFePO_4_ cathode

## Abstract

Lithium (Li) metal is regarded as the ideal anode for rechargeable Li-metal batteries such as Li-S and Li-air batteries. A series of problems caused by Li dendrites, such as low Coulombic efficiency (CE) and a short circuit, have limited the application of Li-metal batteries. In this study, a graphene-modified three-dimensional (3D) Copper (Cu) current collector is addressed to enable dendrite-free Li deposition. After Cu foam is immersed into graphene oxide (GO) suspension, a spontaneous reduction of GO, induced by Cu, generates reduced graphene oxide on a 3D Cu (rGO@Cu) substrate. The rGO@Cu foam not only provides large surface area to accommodate Li deposition for lowering the local effective current density, but also forms a rGO protective layer to effectively control the growth of Li dendrites. As current collector, the rGO@Cu foam shows superior properties than commercial Cu foam and planar Cu foil in terms of cycling stability and CE. The rGO@Cu foam delivers a CE as high as 98.5% for over 350 cycles at the current density of 1 mA cm^−2^. Furthermore, the full cell using LiFePO_4_ as cathode and Li metal as anode with rGO@Cu foam as current collector (LiFePO_4_/rGO@Cu-Li) is assembled to prove the admirable capacities and indicates commercialization of Li-metal batteries.

## Introduction

Electrical energy storage has garnered great attention when considering the problems of pollution and global warming from the burning of fossil fuels and biomass (Goodenough and Kim, 2010). One of the most important candidates is the rechargeable Li battery (Ji et al., 2011; Lu et al., 2014b; Zhao et al., 2015), since it offers higher stored volume and gravimetric energy density (Goodenough and Kim, 2010; Manthiram et al., 2012; Narayanan et al., 2012). Li metal is considered to be a very promising anode material, because it holds quite a higher theoretical specific capacity (3,860 mAh g^−1^), lower density (0.53 g cm^−3^), and a lowest redox potential −3.04 V vs. the standard hydrogen potential electrode) than other anode materials (Xu et al., 2014; Zhao et al., 2015). However, the safety hazards caused by the Li dendrite limit its real applications. Unlike the commercial “host” material, the Li metal anode undergoes the process of Li deposition/stripping during cycling, leading to uncontrollable dendritic Li *via* repeated charging and discharging (Ding et al., 2013; Yun et al., 2016; Li Q. et al., 2017; Ma et al., 2017). When Li is deposited on a planar substrate, the small dendrites are formed first, resulting in an electric field that is distributed unevenly, further promoting the Li^+^ inhomogeneous concentrate (Yun et al., 2016; Li Q. et al., 2017). As the Li dendrites grow, it can render the breakdown/repair of the solid electrolyte interphase (SEI) repeatedly, owing to continuous interfacial reactions (Liu et al., 2017; Gu et al., 2018; Yan et al., 2018). In this way much of the electrolyte is consumed, severely reducing the Coulombic efficiency and capacity decay (Lee et al., 2015; Ye et al., 2017). At the same time, Li dendrites can penetrate the separator further causing an irreversibly infinite volume change and even catastrophic safety hazards (Song et al., 2011; Cheng et al., 2017; Tang et al., 2018).

As noted above, multifarious strategies have been proposed in order to make the Li metal electrodes viable by controlling Li^+^ flux to facilitate uniform deposition of Li and enable formation of a stable SEI (Wu et al., 2017; Li et al., 2018). A variety of electrolyte additives have been employed to modify electrolyte composition and to stabilize the SEI film (Haregewoin et al., 2016). Different kinds of polymeric and ceramic electrolytes have been proven to suppress Li dendrite growth effectively (Kamaya et al., 2011; Kotobuki et al., 2011; Bouchet et al., 2013; Han et al., 2017; Li et al., 2019), however, a series of problems such as chemical instability and low ionic conductivity for application still exists when in contact with Li metal (Li et al., 2015). LiPF_6_ was mixed with LiTFSI-LiBOB dual-salt/carbonate-solvent-based electrolytes which can improve the charging capability and cycling stability of Li-metal batteries notably (Zheng et al., 2017), and which cannot achieve stability with the constant consumption of additives in long-term cycles. Another method to suppress Li dendrite growth is planting an artificial SEI layer by manipulating the formation process (Gao et al., 2017) or inserting an interface protective layer (Lu et al., 2014a; Kozen et al., 2015; Lin et al., 2016; Bobnar et al., 2018; Shi et al., 2019). While, there is not enough mechanical strength of the artificial film to suit large volume expansion in extended periods (Yan et al., 2018). It is reported that a flexible, interconnected, hollow amorphous carbon nanosphere coated on Li metal is desired to accommodate the volumetric expansion of Li deposition without mechanical damage (Zheng et al., 2014). In addition, a composite Li metal anode with an ion-conducting mesoscale skeleton can improve electrochemical performance significantly (Liang et al., 2019). Very recently, the other approach to inhibit Li dendrites by modifying the current collector has been widely discussed. It was found that by creating the CuO nanostructure on the Cu surface as a current collector, exhibits an improved capacity for Li batteries (Liu et al., 2016; So et al., 2018; Zhang et al., 2018). Moreover, the 3D conductive framework as a current collector can suppress the growth of dendrites, because of its large specific surface and low local current density for the Li metal anode (Xie et al., 2016; Yun et al., 2016; Li Q. et al., 2017). However, the pore size of the 3D substrate is thought to have a great impact on electrical conductivity. 3D Cu foam that is too large in size is considered unsuitable for current collectors (Yang et al., 2015).

Based on the above-mentioned discussion, we propose a spontaneous formation of rGO@Cu foam *via* the reaction of 3D Cu foam with graphene oxide to substitute the commercial Cu as a current collector. To prove the excellent properties of the rGO@Cu foam current collector, it was directly used to assemble a half-cell as an electrode with Li metal as the counter electrode. By contrast, 3D Cu foam and 2D planar Cu foil were also directly used separately to assemble batteries with a Li metal sheet as a counter electrode. Compared to three kinds of cells, the rGO@Cu foam inhibits the growth of Li dendrites effectively as well as exhibiting superior properties to 3D Cu foam and planar Cu foil current collectors. The 3D porous structure can reduce the effective electrode current density and provides more space to accommodate Li metal deposition because of its large specific surface. Furthermore, the rGO layer can enhance electrical conductivity and structural stability (Li G. et al., 2017). After cycling, there are minimal dendrites in the 3D Cu foam substrate and the maximum dendrites in the planar Cu foil. Furthermore, the LiFePO_4_/rGO@Cu-Li full cell was fabricated to estimate the advantageous capabilities of the rGO@Cu foam current collector. The remarkable cycling stability and rate performance of the LiFePO_4_/rGO@Cu-Li full cells make it possible to utilize in commercial.

## Experimental Section

### Materials Preparations

**Figure 1:**
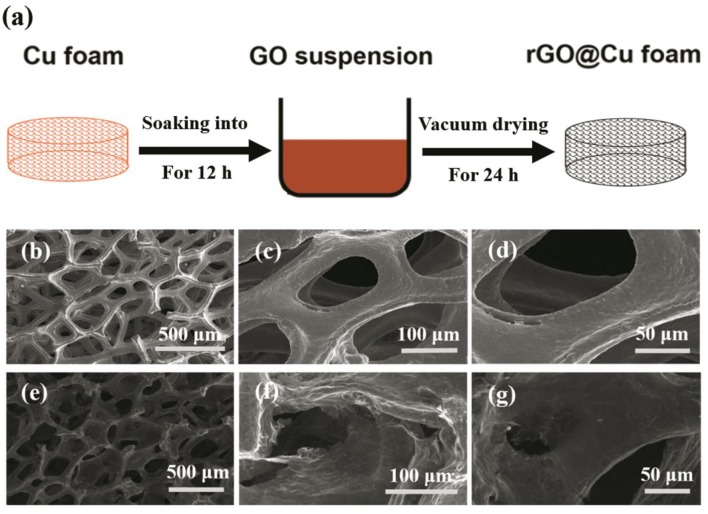
Schematic illustration of the rGO@Cu foam formation process. **(a)** rGO@Cu foam obtained from Cu foam in the 1 mg/ml aqueous GO suspension for 12 h. SEM images of **(b–d)** Cu foam and **(e–g)** rGO@Cu foam.

The schematic diagram for the preparation of the rGO@Cu foam is shown in Figure 1a. To begin this process, GO was prepared from purified natural graphite according to the Hummers' method (Hu et al., 2013; Zaaba et al., 2017; Bai et al., 2018) (details refer to the Supplementary Material). The commercial Cu foam was immersed into the 1 mg/ml graphene oxide (GO) suspension (Figure S1) for 12 h until the black reduced graphene oxide layer covered the whole surface. Then, the Cu foam was removed from the GO suspension and transferred to a vacuum drying oven (DZF-2B) for 24 h, after which, the rGO@Cu foam was obtained.

### Material Characterizations

Morphologies of samples were conducted on a field-emission scanning electron microscope (FESEM, NOVA 450, USA). At the same time, Energy-dispersive (EDS) investigation was collected to analyze element distribution of the sample. X-ray photoelectron spectroscopy (XPS) was chosen to characterize the compound of samples. XPS analysis was conducted with the SDTQ600 Type thermo gravimetric analyzer and the XPS spectra were adopted using monochromatic Al Kα (1486.6eV) X-ray emission-spot size was 650 μm.

### Electrochemical Characterizations

The bare Cu foil, 3D Cu foam and rGO@Cu foam were punched out into discs with a diameter of 12 mm and were tested as different current collectors. To evaluate the properties of repeated Li deposition/stripping in planar Cu foil, commercial Cu foam, and rGO@Cu foam current collectors, CR2016 coin cells were directly assembled using the three kinds of current collectors, with bare Li metal as counter electrodes. The separator was a Celgard-2325 microporous polypropylene film. The electrolyte was a 1 M lithium bis (trifluoromethanesulphonyl) imide (LiTFSI) in 1,3-dioxolane (DOL) and 1,2- dimethoxyethane (DME) (volume ratio 1:1) with 2% lithium nitrate (LiNO_3_) without any additives. Before the symmetrical cell test, the current collectors were first initialized by cycling from 0 to 1 V at 50 μA for five cycles to remove surface contaminations and to stabilize the interface. Then 1 mAh cm^−2^ of Li was plated on the current collector at a current density of 1 mA cm^−2^ and the cutoff potential for the discharge process was set to 1.0 V. To confirm the possibility of a practical application of the modified current collector, the LiFePO_4_ full cell was built using LiFePO_4_ as a cathode and Li metal as an anode with 2D planar Cu foil (LiFePO_4_/2D-Cu-Li), 3D Cu foam (LiFePO_4_/3D-Cu-Li), or a rGO@Cu foam current collector. 1 mAh cm^−2^ of Li was first deposited onto the different Cu current collectors by a half-cell using Li foil as a counter electrode. The cell was then disassembled in an argon-filled glovebox and a new anode was reassembled into a full cell with LiFePO_4_ as the cathode (for details refer to the Supplementary Material). The electrolyte was 1.0 M LiPF_6_ in ethylene carbonate (EC)/dimethyl carbonate (DMC) (1:1 in volume). The LiFePO_4_ full cells were cycled between 2.0 and 4.2 V at 1 C. All the cells were assembled in the argon-filled glovebox and were tested using the LANHE multi-channel battery testing system and PARSTAT 2273 Electrochemical System (Princeton Applied Research, USA).

## Results and Discussion

**Figure 2:**
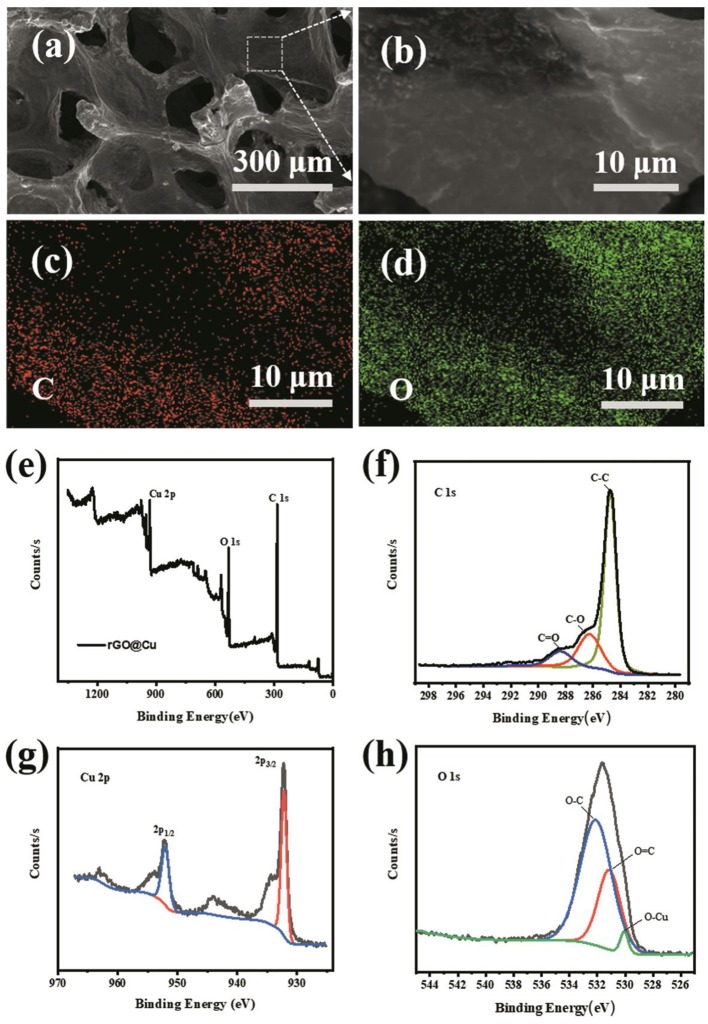
**(a)** SEM images of the morphology of the rGO@Cu foam current collector. **(b)** Magnified view of the rGO from rGO@Cu foam and corresponding element mapping of **(c)** C, **(d)** O. **(e)** XPS spectra of rGO@Cu foam and the corresponding **(f)** C 1s, **(g)** Cu 2p, and **(h)** O 1s peaks.

The pictures of planar Cu foil, bare Cu foam current collector and rGO@Cu foam are shown in Figure S2. A bare Cu foam of reddish brown changed into black after soaking in the GO suspension, showing that the graphene is evenly covered on bare Cu foam. The top view SEM images of commercial Cu foam at different magnifications are shown in Figures 1b–d and rGO@Cu foam is shown in Figures 1e–g. An abundance of pores can be observed in the SEM images of both commercial Cu foam and rGO@Cu foam, confirming the 3D porous structure of samples. Meanwhile, it can be clearly seen that rGO is attached to the Cu skeleton. Figure 2a displays the top view SEM images of a modified Cu foam current collector. Compared with the bare Cu foam, there is a layered structure connected to the Cu skeleton (Figure S3). This layered structure is considered to be rGO because the GO is spontaneously reduced by Cu, and Cu is oxidized to form CuO. The SEM image of the surface of the layered structure is magnified on Figure 2b and the corresponding elemental mappings are shown in Figures 2c,d. It can be discovered that only C (Figure 2c) and O (Figure 2d), as major elements, are distributed evenly and independently on the layered structure. To further illustrate the composite of Cu foam with GO, XPS spectra of samples are collected in Figures 2e–h. Figure 2e exhibits the XPS survey spectra measured in the range of binding energies from 0 to 1,361 eV and the peaks of C 1s, O 1s, and Cu 2p can be clearly observed. As shown in Figure 2f, the intensities of the C-O (286.25 eV) and C = O (288.38 eV) functional groups are significantly decreased compared with the spectra of GO (Stankovich et al., 2007; Luo et al., 2011; Kim et al., 2013). At the same time, the main peak at 284.75 eV indicates that most of the C-C functional group was formed (Stankovich et al., 2007). This result can be explained by the fact that GO is reduced to rGO. The high-resolution scan of Cu 2p is displayed in Figure 2g, a double peak with binding energies at 932.75 and 952.75 eV is attributed to Cu 2p3/2 and Cu 2p1/2 of Cu^2+^ in CuO, respectively. Also, the gap between the Cu 2p3/2 and Cu 2p1/2 energy levels is 20.0 eV, corresponding to the split orbit for Cu^2+^ (Soleimani and Moghaddami, 2017). These results can be considered to show that the Cu was oxidized to CuO. To further prove the existence of oxygen-containing functional groups and CuO, the O 1s core-level spectrum is shown in Figure 2h. There are mainly three peaks centered at 532.88, 531.82, and 530.7 eV, which is approximately consistent with the O-C, O = C, and O-Cu (Novakov and Prins, 1971; Mattevi et al., 2009). The peaks of O 1s of the sample are significantly increased compared with GO, indicating that most of the oxygen functional groups are reduced and the rGO is formed (Kim et al., 2013). In addition, the existence of O-Cu further proves the formation of CuO. As a result, the existence of rGO and CuO without any external conditions proves that the spontaneous reaction is real.

**Figure 3:**
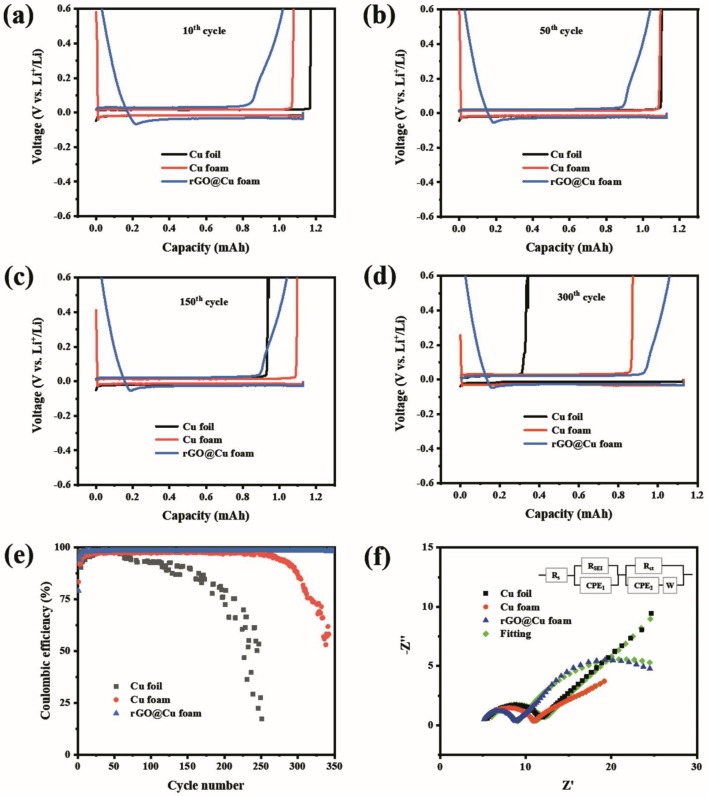
The voltage profiles for the **(a)** 10th, **(b)** 50th, **(c)** 150th, and **(d)** 300th cycles with 2D planar Cu, 3D commercial Cu foam, and rGO@Cu foam as an electrode and Li metal as a reference electrode at a current density of 1.0 mA cm^−2^. **(e)** CE of cells using Cu foil, Cu foam, or rGO@Cu foam as an electrode with Li metal as a counter electrode at a current density of 1 mA cm^−2^ with the deposition capacity at 1 mAh cm^−2^. **(f)** Nyquist plots of Cu foil, Cu foam, and rGO@Cu foam. The impedance is obtained after 300 cycles with a current density at 1 mA cm^−2^ and a cycling capacity of 1 mAh cm^−2^.

The electrochemical performance of the coin cells with 2D planar Cu foil, 3D commercial Cu foam, and rGO@Cu foam as an electrode with Li metal as a counter electrode is displayed in Figure 3. The coin cells with three types of current collectors were first cycled five times from 0 to 1 V at 50 μA to remove surface impurities and to help form a stable SEI film. Figures 3a–d exhibit the galvanostatic cycling voltage profiles on the 2D planar Cu foil, 3D commercial Cu foam, and rGO@Cu foam electrode for cycles at 10th, 50th, 150th, and 300th. After the initial 10 cycles, 2D planar Cu foil and 3D commercial Cu foam display a higher discharge voltage plateau and a lower charge voltage plateau than rGO@Cu foam (Figure 3a), indicating a much lower polarization of planar Cu foil and 3D Cu foam compared to rGO@Cu foam. This might be attributed to the fact that the surface of rGO@Cu foam was not activated completely by the electrolyte, causing the accumulation of Li^+^ on the current collector. Notice that this phenomenon disappears after fifty cycles (Figure 3b). In addition, compared with the 50th (Figure 3b), 150th (Figure 3c), and 300th (Figure 3d) cycles, a significant capacity decay of planar Cu foil is observed, which may be due to the growth of Li dendrites further leading to the formation of dead Li and the repeated consumption of the electrolyte caused by unstable SEI. For commercial Cu foam, the capacity decay appears about 300 cycles, illustrating that there is less irreversible Li deposition on the 3D Cu and more stable SEI than planar Cu. By contrast, the galvanostatic charge/discharge profiles of the rGO@Cu foam shows outstanding stability even after 300 cycles, and rGO@Cu foam also exhibits a superior capacity retention. These results demonstrate that the deposition of Li on rGO@Cu foam is more uniform and the formation of SEI is more stable than planar Cu and commercial Cu foam. To evaluate the long-term cycling stabilities of the Li anode with the three types of current collectors, the symmetric cells were assembled and examined at a current density of 1 mA cm^−2^ with a capacity of 1 mAh cm^−2^. As shown in Figure S4, after 84 h, an abrupt voltage drop is detected for the planar Cu foil current collector with fluctuating voltage in the later hours. This could be explained by an internal soft short-circuit with Li dendrite penetration (Lin et al., 2016). A distinct decrease in polarization is observed in commercial Cu foam, in the initial 80 h, may be due to the gradual stabilization of the SEI layer. By contrast, the 3D rGO@Cu current collector maintains a much lower and more stable hysteresis after 400 h, illustrating that its extended reduced graphene layer can reduce the local current density and inhibit the growth of Li dendrites. The cycling Coulombic efficiencies of 2D Cu foil, 3D commercial Cu foam, and rGO@Cu foam current collectors were examined at a current density of 1 mA cm^−2^. CE is computed by the ratio of the total amount of Li stripped away, vs. the deposited amount on the current collector in each cycle. As shown in Figure 3e, the CE of 2D Cu foil is stabilized at 97% during the 60 cycles, and then the CE is reduced gradually to <92% after 110 cycles. This may be ascribed to the irreversible Li deposition because of the formation of Li dendrites and dead Li. As for the commercial Cu foam current collector, the CE can reach more than 97% and be sustained during 225 cycles, showing that the Cu foam current collector with a special porous structure does help to accommodate more Li^+^, hence hindering the growth of Li dendrites and extending the cycle life, whereas the CE of the 3D Cu foam becomes unstable after only 260 cycles and drops to below 96%. The rGO@Cu foam as current collector was conducted to further develop the cycling performance in comparison to 2D planar Cu foil and commercial Cu foam. The Columbic efficiency of rGO@Cu foam maintains a stability as high as 98.5% for over 350 cycles, which demonstrates that the covered reduced graphene oxide on the 3D Cu foam has significant effects and improves the Columbic efficiency and cycling stability in contrast to commercial Cu foam. To further explain the effect of the reduced graphene oxide layer on the improvement of the Cu foam interfaces, EIS was elected to evaluate the interfacial resistance of the planar Cu foil, commercial Cu foam, and rGO@Cu foam current collectors. The Nyquist plots of the three types of current collectors is displayed in Figure 3f. Comparing the intercept and the diameter of the first semicircle in each sample, the rGO@Cu foam possesses smaller SEI film resistance (4 Ω cm^2^) than commercial Cu foam (5.9 Ω cm^2^) and planar Cu foil (6.5 Ω cm^2^), which could be attributed to the stable SEI film modified by the reduced graphene layer. In addition, the second semicircle reflects the charge transfer (interfacial) resistance Rct (Schipper et al., 2018; Wu et al., 2019). The rGO@Cu foam and commercial Cu foam have considerably smaller charge transfer resistance (24 and 30 Ω cm^2^) compared with planar Cu foil (184 Ω cm^2^). It suggests that a large amount of Li dendrites and dead Li are deposited on the surface of Cu foil. All the results confirm that the rGO@Cu foam current collector possesses superior properties such as higher electrochemical stability, better capacity retention, and Li dendrite suppression.

**Figure 4:**
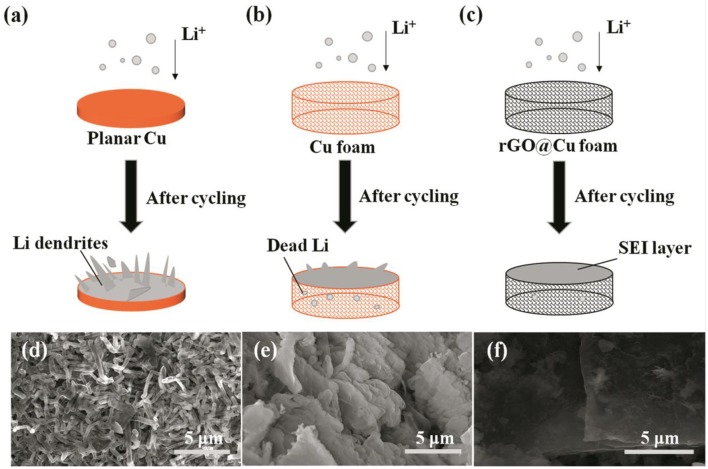
Schematic representations showing the process of Li deposition on **(a)** bare planar Cu, **(b)** Cu foam, and **(c)** rGO@Cu foam. SEM images of surfaces of **(d)** bare planar Cu, **(e)** Cu foam and **(f)** rGO@Cu foam after cycles.

Figure 4 illustrates the behaviors of Li deposition on the 2D planar Cu, 3D commercial Cu foam, and rGO@Cu foam current collectors. Li is deposited on the substrate and unavoidably form some protuberances on the surface of planar Cu foil (Figure 4a), leading to the electric field being distributed unevenly, further promoting Li accumulation at the protuberances, finally growing into Li dendrites or even forming “dead Li.” On the other hand, the formation of Li dendrites destroys SEI and further leads to the repeated formation of SEI film. Furthermore, these problems could deteriorate during cycling. In a Cu foam current collector (Figure 4b), owing to its special 3D porous structure, more space could be provided to accommodate Li. The porous current collector exhibits lower interfacial resistance and local current density compared to planar Cu, therefore Li^+^ will distribute homogeneously on the surface of the 3D Cu foam and few Li dendrites will be formed. However, the phenomenon of Li dendrites still worsens after cycling. For the rGO@Cu foam current collector (Figure 4c), Li dendrites are restrained effectively. The rGO@Cu foam current collector displays outstanding electric conductive and mechanical strength for Li deposition. Li favors deposition on the inner surface of the rGO@Cu foam current collector. This obviously strengthens the stability of SEI and reduces the consumption of Li. In order to clearly observe the morphology of Li dendrite growth, the SEM images of the surfaces of current collectors are shown in Figures 4d–f with high magnifications. In the planar Cu foil, many Li filaments (Figure 4d) with lengths of <3 μm (Figure S5a) could be discovered after long-term cycling, leading to penetration of the separator and causing short circuiting of the cell. In addition, a loose structure is composed as a result of the formation of Li dendrites and dead Li. This loosely aggregated structure further gives rise to the electrical field being distributed unevenly and accelerates Li being deposited unequally. By contrast, for a 3D commercial Cu foam, there are only a few fibrous Li and some mossy Li formed on the surface of the current collector (Figure 4e), indicating that the porous structure of the 3D Cu foam current collector revealed an excellent property to accommodate the expansion of the deposited Li. Figure S5b shows a magnified surface of 3D Cu foam after cycling, illustrating that it provides a more stable working electrode structure and interface than planar Cu foil. Moreover, compared with commercial Cu foam, the SEM images of rGO@Cu foam, as shown in Figure 4f and Figure S5c, clearly show that the surface of this composite electrode is remarkably smooth in general without obvious Li dendrites or mossy Li under the same conditions. This result may be explained by the fact that the rGO@Cu foam current collector can prevent the growth of Li dendrites and remove the potential of Li-metal battery hazards. This flat surface could be due to the rGO@Cu foam combined with the benefits of the rGO and 3D porous structure. rGO works as a protective layer to inhibit the growth of Li dendrites and the 3D porous structure promotes uniform deposition of Li into the interspace of the rGO@Cu foam current collector, improving cycling stability and the lifespan of Li-metal batteries.

**Figure 5:**
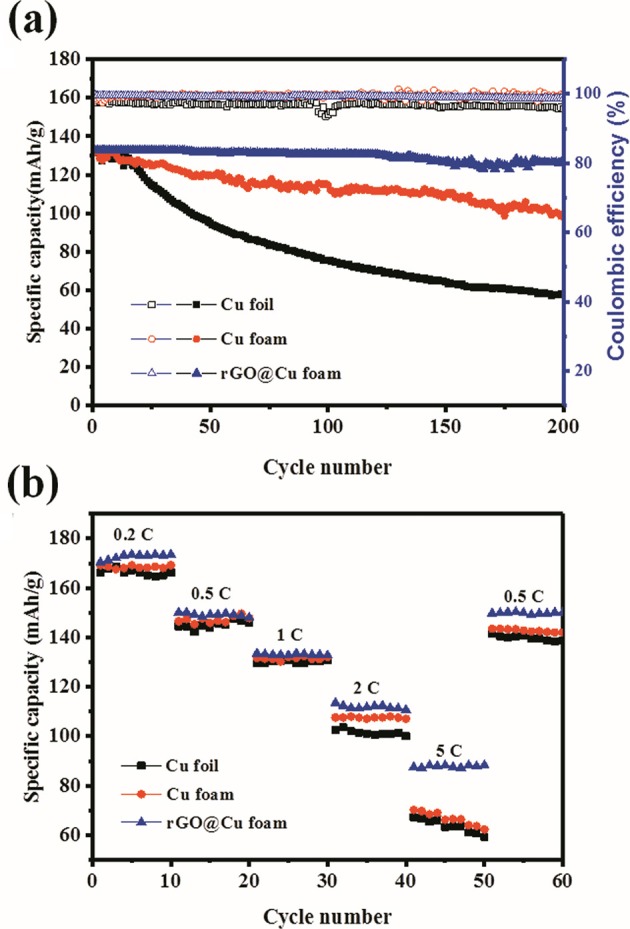
Electrochemical characterization of full cells using a LiFePO_4_ cathode and a Li metal anode with 2D planar Cu, 3D Cu foam, or rGO@Cu foam as a current collector. **(a)** Cycling performance of LiFePO_4_/2D-Cu-Li, LiFePO_4_/3D-Cu-Li, LiFePO_4_/rGO@Cu-Li at 1 C. **(b)** Rate performance of LiFePO_4_/2D-Cu-Li, LiFePO_4_/3D-Cu-Li, LiFePO_4_/rGO@Cu-Li from 0.2 to 5 C.

To further investigate the battery performance under practical applications, the LiFePO_4_/2D-Cu-Li, LiFePO_4_/3D-Cu-Li, and LiFePO_4_/rGO@Cu-Li full cells were assembled and galvanostatically cycled from 2.0 to 4.2 V at 1 C. Figure 5a exhibits the long-term cycling performance of the LiFePO_4_/2D-Cu-Li, LiFePO_4_/3D-Cu-Li, and LiFePO_4_/rGO@Cu-Li full cells at 1 C. For LiFePO_4_/2D-Cu-Li cells, the discharge capacity is about 132.2 mAh g^−1^ in the first cycle and starts to decrease obviously after 15 cycles. The reversible capacity of LiFePO_4_/2D-Cu-Li cells is dropped to 57.6 mAh g^−1^ with a CE of 95.7% at 200 cycles. The depletion of the electrolyte is due to the continuous decomposition at Li metal and the formation of “dead Li.” While as for the LiFePO_4_/3D-Cu-Li cells, the reversible capacity degrades gradually from the beginning with 131.7 to 98.6 mAh g^−1^ and the CE is maintained at about 99% during 200 cycles. It can be seen that the cycling performance of LiFePO_4_/3D-Cu-Li cells is greatly improved compared with LiFePO_4_/2D-Cu-Li. By contrast, the LiFePO_4_/rGO@Cu-Li cells exhibit a higher initial capacity of 132.9 mAh g^−1^ and better capacity retention with a discharge capacity of 126.2 mAh g^−1^ after 200 cycles. Moreover, it shows a more stable CE of 99% for more than 200 cycles, illustrating that the LiFePO_4_/rGO@Cu-Li cells display outstanding capacity retention and cycling stability. Figure 5b shows the rate performance of these three kinds of LiFePO_4_ full cells with 2D Cu, 3D Cu foam, and rGO@Cu foam current collectors under different current densities. The rate capability of cells was evaluated under cycling at rates varying from 0.2 C to 5 C with intervals of 10 cycles. It can be observed that the capacity almost goes back to the previous corresponding value when the rate is recovered to 0.5 C, and all the full cells display obvious capacity decay with current density increases. The LiFePO_4_/rGO@Cu-Li cells exhibit a capacity of 88 mAh g^−1^ at 5 C, indicating its enhanced discharge capacity, especially at a high cycling rate. While under the same conditions, the capacity of LiFePO_4_/2D-Cu-Li cells almost reach 67 mAh g^−1^ and LiFePO_4_/3D-Cu-Li cells are 70 mAh g^−1^, which is still less than the LiFePO_4_/rGO@Cu-Li cells. In addition, the LiFePO_4_/rGO@Cu-Li cells always remain at the highest capacity compared to LiFePO_4_/2D-Cu-Li and LiFePO_4_/3D-Cu-Li at different current densities. These results indicate that the LiFePO_4_/rGO@Cu-Li shows excellent rate performance and a higher capacity, demonstrating its great potential for practical application.

## Conclusions

In summary, in this study, we have reported a simple but effective strategy to suppress the growth of Li dendrites and to facilitate the uniform deposition of Li using a rGO@Cu foam as a current collector. After soaking in GO suspension, the commercial Cu foam is covered with a layered structure of rGO. The rGO@Cu foam exhibits superior performance because the rGO layer works as a protective film to inhibit the growth of Li dendrites. Furthermore, its special porous structure can provide more space to accommodate Li metal deposition and reduces the effective electrode current density. The half-cell using rGO@Cu foam as an electrode and Li metal as a counter electrode exhibits a high CE of above 98.5% after 350 cycles under the current density of 1 mA cm^−2^. As for the LiFePO_4_/rGO@Cu-Li full cells, the reversible capacity is maintained at 126.2 mAh g^−1^ and the CE can remain as high as 99% after 200 cycles at 1 C. The LiFePO_4_/rGO@Cu-Li full cells also show a stable rate performance compared to LiFePO_4_/2D-Cu-Li and LiFePO_4_/3D-Cu-Li cells. The superior properties of the rGO@Cu foam current collector are beneficial in achieving the effective suppression of Li dendrites for next-generation Li-metal battery applications.

## Data Availability Statement

All datasets generated for this study are included in the article/Supplementary Material.

## Author Contributions

All authors contributed to manuscript revision, read, and approved the submitted version.

### Conflict of Interest

The authors declare that the research was conducted in the absence of any commercial or financial relationships that could be construed as a potential conflict of interest.
