# Supplementary material for: Graphene-Modified 3D Copper Foam Current Collector for Dendrite-Free Lithium Deposition
Source: Front Chem. 2019 Nov 27;7:748. doi: 10.3389/fchem.2019.00748 (PMC6890847; doi:10.3389/fchem.2019.00748)
Supplement: Supplementary file 1 [file Data_Sheet_1.pdf]

Supplementary Material

Preparation of graphene oxide (GO) suspension. GO was obtained using a modified Hummers' method from pure graphite powder. In this method, to begin with, sulfuric acid (H_2SO_4) and phosphoric acid (H_3PO_4) were mixed at a volume ratio of 9:1 under stirring. Then 0.225 g of pure graphite powder was added into the liquid mixture and stirred constantly. After that, 1.32 g of potassium permanganate ($KMnO_4$) was dropped in succession. The mixture turned dark green under stirring conditions for 6 h. 0.675 ml of hydrogen peroxide (H_2O_2) was added slowly to reduce the residual oxidant with stirring for 10 min. After cooling, 10 ml of hydrochloric acid (HCl) and 30 ml of deionized water (DIW) was added to the mixture. The product was transferred to a centrifuge at 5000 rpm for 7 min (TG20-WS). The residuals were obtained after the process of filtration and rewashed with HCl and DIW three times. The GO solution was dried using an oven at 90°C for 24 h to produce the powder of GO. The 1 mg/ml GO suspension was achieved *via* GO dissolved in DIW with a volume ratio of 1:1 under ultrasonic (KQ-100DE) for 10 min.

Figure S1. Pictures of the change of Cu foam in the 1 mg/ml GO suspension for 12 h.

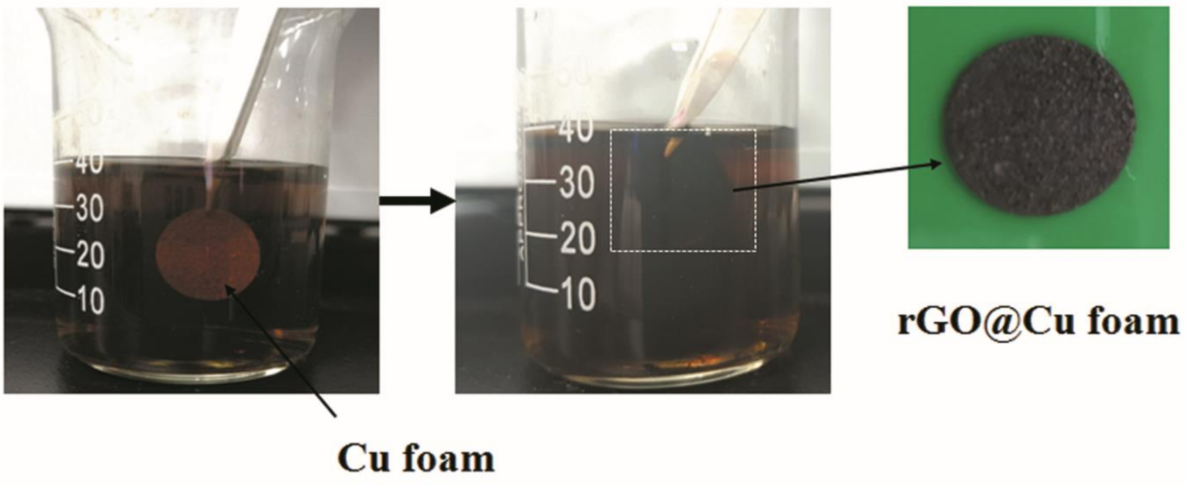

Preparation of rGO@Cu foam. As shown in Figure S1, the commercial 3D Cu foam was immersed in 1 mg/ml GO suspension for several hours, the 3D Cu foam begun to blacken locally for a while and completely turned black after 12 h. At this point, GO was reduced to rGO and Cu was oxidized to Cu^{2+}.

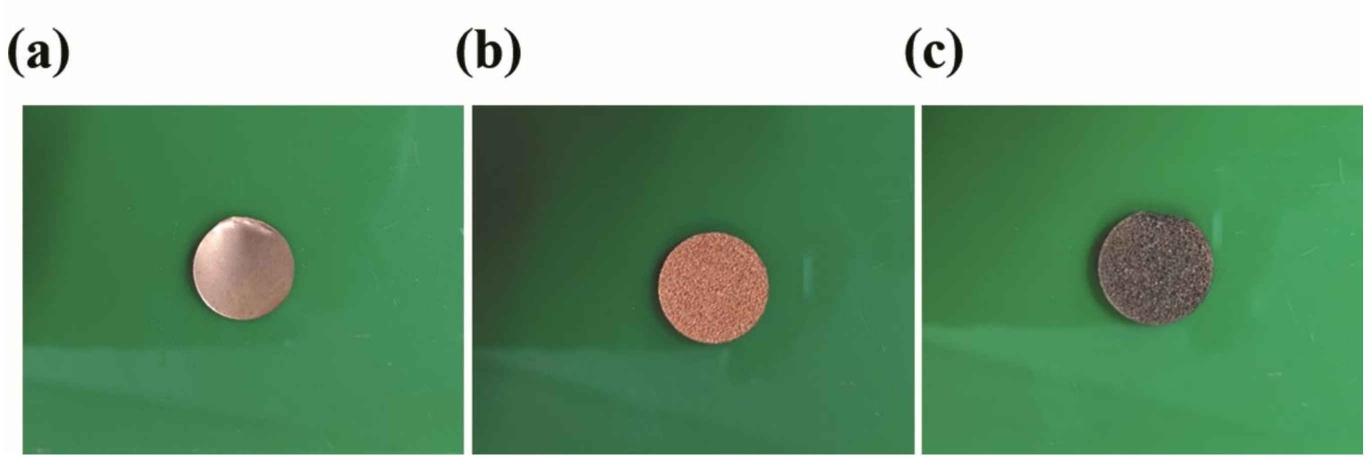

Figure S2. Pictures of the morphology of (a) planar Cu foil, (b) 3D Cu foam, and (c) 3D rGO@Cu foam.

Preparation of $LiFePO_4$ cathode. $LiFePO_4$, super P and poly (vinylidene difluoride) (PVDF) were mixed in a mass ratio of 8:1:1, stirring for 10 min. The solid mixture was stirred continuously with N-methyl-2-pyrrolidone (NMP) for 30 min. Then the slurry was coated on Al foil and dried at 60°C under vacuum for 24 h. The Al foil coated with $LiFePO_4$ was cut into discs with a diameter of 12 mm and then the required cathode was prepared.

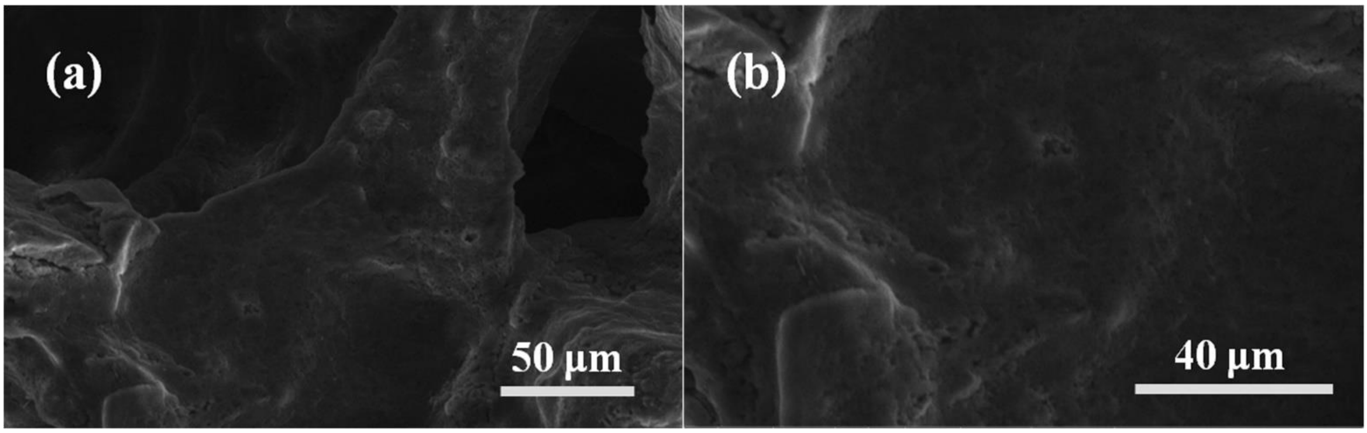

Figure S3. SEM of the morphology of 3D rGO@Cu foam. (a) The surface of 3D Cu skeleton. (b) The surface of layered structure.

As shown in Figure S3a, the Cu skeleton is covered with a layered structure. Figure S3b shows an enlarged surface of rGO, indicating that rGO is connected with Cu skeleton.

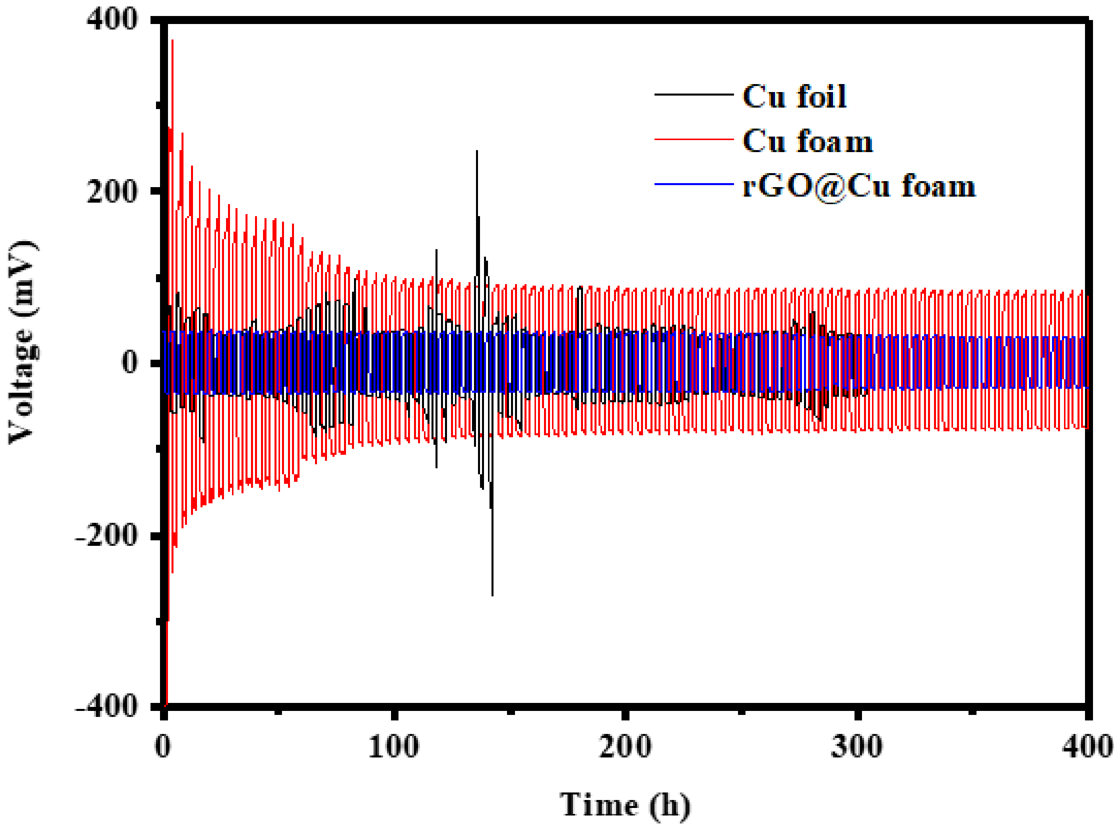

Figure S4. Voltage profiles of symmetric cells with a capacity of 1 mAh cm^{-2} at a current density of 1 mA cm^{-2}.

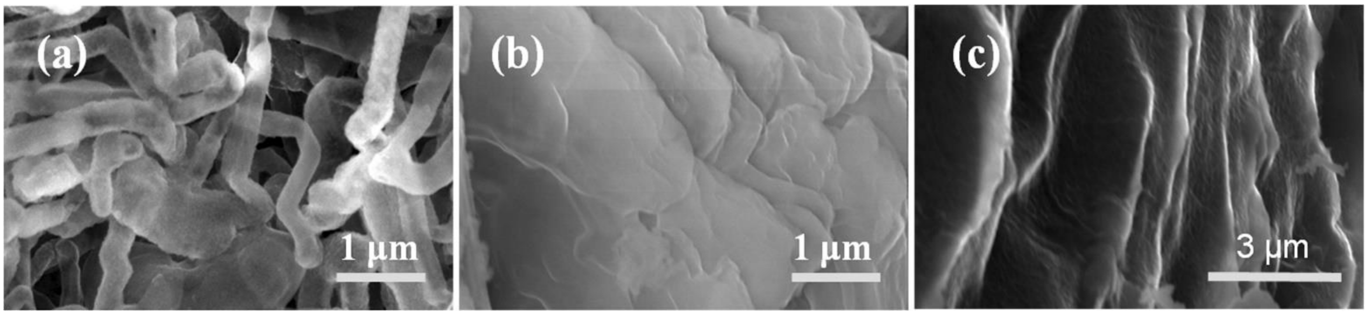

Figure S5. The SEM of the morphology characterization of Li deposited onto (a) planar Cu foil, (b) 3D Cu foam and (c) rGO@Cu foam.
